# Authorship and Authority

**Published:** 1911-04-15

**Authors:** 


### Authorship and Authority.

The periodical criticism of the examination system as a test of merit has just come round again. In medicine, a good deal perhaps depends on examinations; because of the fact that nearly all distinguished medical men hold high qualifications, one seldom hears the question raised. Many hospital appointments, again, are confined to certain graduates and diplomates. To some extent medical journals are open to all. The resulting heap of literature is much too big, and shows far too much dross and very little ore, it is true, but no one can say truly that the only offenders are those who, if some had their way, would be constituted into a "lower branch" of the profession.

The list of original contributions to European or better-class American medical periodicals mostly consists, firstly, of a paper by some famous old *emeritus* or of a report of some set oration or lecture. Then come articles by well-known physicians or surgeons or sanitarians or pathologists, articles essential, fairly often, to students of the subject treated of, but which may be just thinly disguised puffs of the writer's professional ability, real or pretended. At the end are crowded in the efforts of youth or of those not high up the ladder of contemporary professional fame. These, to be candid, are mostly without value, but there is a residue of two or three a year which will live, albeit at first and for a considerable period in a state of suspended animation. For the sake of the last, as also on the principle of the "first bite," and to conciliate subscribers' opinion, it is customary to show a liberal attitude; and we think this course not only large-minded but also wholly wise. Coercive measures would however be voted for by some who endorse Oppenheimer's impatient written comment—"Weg mit Dissertationen und Festschriften!"

Open opposition of that kind is rare in this smoother-spoken country; but private opinion in certain quarters here is very similar. In a recent conversation, the topic of which was a short article by an undistinguished writer raising a novel and important point, it was said that he would do well to secure a higher qualification. Some objection or other was raised, whereupon the first speaker—not, to be sure, an investigator himself, but usually the most diplomatic and conciliatory of men, exclaimed angrily—"Well, he mustn't expect much notice to be taken of what he says unless he does!" Obscurantism like this, however, must surely be exceptional. None the less, philosophic note has to be taken of it, as of any other phenomenon. The ordinary "consultant" has doubtless never very far from his mind the delights scorned, the fees paid, and the laborious days of his student life, as also a clear remembrance of the precariousness of his early career. As others—so far—have had the profit, he thinks that the honour should be his; and here indeed he might quote from the "Ethics" in confirmation. In a good many cases he is right, but the whole history of discovery shows that he may be wrong, and then the consequences of his mistake are overmuch to be atoned for by any number of previous just decisions.

